# Implementation of a Multidisciplinary Rounding Tool in a Medical Intensive Care Unit and Its Association with Rounding Efficiency and Multidisciplinary Collaboration

**DOI:** 10.3390/jcm15197437

**Published:** 2026-09-24

**Authors:** Natalie C. Washburn, Todd A. Walroth, Arghyadeep Ganguly, Thomas Blostica, Akram Alnounou, Chelsea Zambrano

**Affiliations:** 1 Department of Pharmacy, Bronson Methodist Hospital, Kalamazoo, MI 49007, USA; 2 Department of Pharmacy, Eskenazi Health, Indianapolis, IN 46202, USA; 3 Department of Internal Medicine, Western Michigan University Homer Stryker M.D. School of Medicine, Kalamazoo, MI 49008, USA

**Keywords:** rounding checklist, ICU Liberation bundle, ABCDEF, multidisciplinary participation, rounding time, efficiency

## Abstract

**Background**: Multidisciplinary rounds are essential for high-quality intensive care but are frequently hindered by inconsistent team participation and logistical inefficiencies. We evaluated the associated impact of implementing a standardized multidisciplinary rounding checklist and real-time paging system on multidisciplinary team attendance, rounding efficiency, and selected clinical outcomes in the ICU. **Methods**: A retrospective, observational pre- and post-intervention cohort study featuring a concurrent control group was conducted at a tertiary care hospital. The study included 331 adults admitted to the medical ICU (MICU) and neurovascular critical care unit (NCCU; control). The intervention combined a standardized rounding checklist incorporating the ICU Liberation (ABCDEF) bundle and FAST HUGS BID mnemonic with a real-time paging notification system to alert team members of round start times and locations. The primary outcome was change in multidisciplinary team participation at the start and end of rounds; secondary outcomes included rounding time per patient and exploratory care-process outcomes. **Results**: Multidisciplinary participation at the start and conclusion of rounds significantly improved for both the MICU advanced practice provider (APP) team (*p* = 0.003 and *p* = 0.002, respectively) and the resident team (*p* = 0.006 and *p* < 0.001, respectively), while attendance in the NCCU group remained unchanged. Median rounding time per patient decreased by 20.8% (13.0 to 10.3 min, *p* < 0.001). In a survivor-only sensitivity analysis, the streamlined workflow decreased the median time to physical and occupational therapy consultations from 4 to 2 days (*p* ≤ 0.002). Invasive device stewardship significantly improved; central venous catheter utilization decreased from 58.0% to 25.0% (*p* < 0.001), and arterial line utilization fell from 55.0% to 26.5% (*p* < 0.001), with median arterial catheter duration decreasing from 5 to 3 days (*p* = 0.001). **Conclusions**: Implementing low-cost, reliable workflow standardization tools was associated with significantly improved multidisciplinary participation and shorter rounding time per patient. Favorable changes in selected care-process measures were also observed, although patient-level clinical outcomes should be interpreted as exploratory and future studies are warranted.

## 1. Introduction

Multidisciplinary rounding is at the core of high-quality care in intensive care units (ICUs). It is the bedrock of facilitating effective communication between healthcare workers, enabling them to make informed and coordinated decisions for care of critically ill patients [1,2]. A systematic review of 43 applicable studies conducted by Lane and colleagues in 2013 found structured multidisciplinary rounds with standardized checklists, wherein each provider has explicitly defined roles, improved patient outcomes, provider satisfaction, and adherence to practice guidelines [2]. A retrospective pre-/post-implementation study conducted by Brown and colleagues in 2020 demonstrated that by implementing a standardized rounding template, as well as a rounding start time and location, rounding time was reduced by 25% and presence of key multidisciplinary providers (e.g., case managers, dietitians, and respiratory therapists) at rounds improved [3].

The Society of Critical Care Medicine (SCCM)’s ICU Liberation Campaign highlights the importance of multidisciplinary team rounds in implementing the ABCDEF bundle (Assessment, Prevention and Management of Pain; Both Spontaneous Awakening and Spontaneous Breathing trials; Choice of Analgesia and Sedation; Delirium Assessment, Prevention and Management; Early Mobility and Exercise, Family Engagement and Empowerment). The ABCDEF bundle has been associated with a reduction in mortality, duration of mechanical ventilation, and ICU readmissions [1,4].

There have been numerous challenges in the implementation of multidisciplinary rounds across sites despite their demonstrated and perceived benefits [2]. Inconsistent rounding start times and locations, unpredictable and variable team member participation, lack of standardized communication, and prolonged rounding duration are common barriers, among others [2,3]. In larger academic medical centers, these challenges tend to be more pronounced given the presence of several specialty intensive care units and multiple teams with personnel sharing across units. Limited literature supports standardized rounding processes reducing rounding time and improving multidisciplinary attendance [3]. However, limited data exists regarding the impact of standardized multidisciplinary rounds on clinical patient outcomes. A large 2016 study led by Cavalcanti and colleagues in Brazil found there was no significant difference between the intervention (*n* = 3327) and control (*n* = 3434) groups with regard to important patient outcomes like in-hospital mortality, central line-associated bloodstream infections (CLABSI), ventilator-associated pneumonia (VAP), urinary tract infection (UTI), ICU length of stay, hospital length of stay, ventilator-free days and head of bed elevation greater than 30 degrees; however, the intervention group did display improvement with regard to usage of lower tidal volumes, avoidance of heavy sedation, and use of central venous and urinary catheters [5]. In a 2020 study of 58 patients, the implementation of standardized multidisciplinary rounds significantly decreased both ICU length of stay and the placement of central venous and arterial lines. Furthermore, the intervention led to a twofold increase in the availability of patient disposition plans. Beyond these clinical metrics, the standardized approach increased the frequency of updates to patients’ families and made non-physician team members feel more valued [6].

At Bronson Methodist Hospital, multidisciplinary team members including respiratory therapists, registered dietitians, pharmacists, social workers, and case managers are shared between three critical care teams, namely the Neurovascular Critical Care Unit (NCCU) team, the Medical ICU Advanced Practice Provider (APP) team, and the Medical ICU Resident team. Before the intervention, these teams conducted rounds at inconsistent times and starting locations with variable durations, leading to suboptimal multidisciplinary participation and several other inefficiencies linked to patient care. Therefore, we undertook this quality improvement study to determine if implementing a standardized rounding checklist with a real-time paging system notification would increase multidisciplinary participation, rounding efficiency, and be associated with improved selected patient outcomes. Our study is the first to our knowledge to explore rounding duration, multidisciplinary participation, and patient outcomes in a pre- and post-implementation manner, paired with a control group.

## 2. Materials and Methods

### 2.1. Study Design

This was a single-center, retrospective, cohort study with a pre- and post-intervention phase with a concurrent nonintervention control group conducted at Bronson Methodist Hospital (BMH), a 434-bed tertiary care medical center in Kalamazoo, MI, USA. The reporting of this study conforms to the Strengthening the Reporting of Observational Studies in Epidemiology (STROBE) statement for observational cohort studies.

### 2.2. Study Population

The study involved non-pregnant patients aged 18 years and above, admitted to the Medical Intensive Care Unit (MICU), Neurovascular Critical Care Unit (NCCU), or intermediate care unit under the neurovascular critical care or medical intensive care teams from 22 September 2021 to 15 December 2023.

### 2.3. Outcomes

The primary outcome was multidisciplinary participation at the start and end of rounds. Secondary outcomes included total rounding duration and rounding time per patient. Patient-level outcomes, including time to Physica therapy (PT)/Occupational therapy (OT) consultation, delirium documentation, invasive device use, ICU mortality, and in-hospital mortality, were considered exploratory care-process and clinical outcomes.

### 2.4. Study Period

The pre-intervention period extended from 22 September 2021 to 5 November 2021. Rounding data from these thirty-three days were collected. The intervention (as described below) was performed from 19 September 2023 to 27 October 2023. Team member education, inauguration of a paging system, and rounding checklist refinements were implemented during this period. The post-intervention period lasted from 30 October 2023 to 15 December 2023, yielding thirty-four days of rounding data to match the duration of pre-intervention data collection. The MICU Resident and Advanced Practice Provider (APP) teams served as the main subjects of the intervention group, whereas the NCCU team was a concurrent control group who did not implement the intervention during the study period.

### 2.5. Intervention

The two primary components of the intervention included (1) the implementation of a standardized multidisciplinary rounding checklist incorporating the ICU Liberation ABCDEF bundle elements and the FAST HUGS BID (Feeding, Analgesia, Sedation, Thromboprophylaxis, Head of Bed Elevation, Ulcer Prophylaxis, Glycemic Control, Spontaneous Breathing Trials, Bowel Function, Indwelling Catheter Removal, De-escalation of Antibiotics) mnemonic; and (2) the creation of a paging system to alert multidisciplinary team members to the start time and location of rounds. The rounding card defined each team member’s role explicitly. The bedside nurse reported overnight events and general patient concerns. The Resident Physician or APP followed up with a systems-based assessment encompassing neurologic (including Richmond Agitation-Sedation Scale [RASS], pain scores, confusion assessment method [CAM] scores), respiratory (including spontaneous breathing trial eligibility), cardiovascular, gastrointestinal, nutritional, renal, hepatic, endocrine, hematologic, infectious diseases, and prophylaxis domains. Multidisciplinary team members including the respiratory therapists (RTs), clinical dietitians, pharmacists, medical social workers, and case managers contributed relevant discipline-specific assessments and plans at designated points during the patient presentations. The teams were educated regarding the intervention on 19 September 2023, and the paging system was launched on 25 September 2023. The rounding card underwent revisions from 25 September to 27 October 2023, based on feedback received from various team members (Figure 1).

### 2.6. Data Collection

For the pre- and post-intervention phases, clinical outcomes data were retrospectively extracted from the electronic medical record (EMR). Pre- and post-intervention rounding data were prospectively collected by the pharmacist team members, who recorded the team members from each discipline present at the start and end of rounds, rounding start and end times, and the number of patients seen during rounds. Standardized illness severity scores (e.g., SOFA, APACHE, etc.) were not available for all patients and therefore not included in our analysis.

### 2.7. Statistical Analysis

The Anderson–Darling test was used to assess normality. Continuous variables were reported as mean ± standard deviation (SD) in case of data with normal distribution and median [interquartile range, IQR] in the case of data with non-normal distribution. Further, categorical variables were reported in numbers and percentages. Inter-group comparisons were done using the Student’s *t*-test or the Mann–Whitney U test for continuous variables and the chi-square test or Fisher’s exact test for categorical variables. Based on the days available in each study period, a convenience sample was used. Statistical significance was pre-defined as *p* < 0.05, and analyses were performed using Minitab Statistical Software (version 21.1, Minitab LLC, State College, PA, USA).

## 3. Results

### 3.1. Patient Demographics and Mortality

A total of 331 patients were included: 131 in the pre-intervention period and 200 in the post-intervention period. The median [IQR] age was similar between pre- and post-intervention groups (65 [50, 75] vs. 63 [52, 75] years, *p* = 0.631). The pre-intervention group had a significantly higher proportion of COVID-19 patients (15.3% vs. 5.0%, *p* = 0.003). Median [IQR] ICU length of stay was similar between the pre- and post-intervention groups (2 [1, 6] vs. 2 [1, 3] days, *p* = 0.200). The ICU mortality (35.9% vs. 11.0%, *p* < 0.001) and in-hospital mortality (49.6% vs. 15.5%, *p* < 0.001) were both significantly lower in the post-intervention group. Despite adjustment for COVID-19 status, ICU mortality (33% vs. 11%, *p* < 0.001) and in-hospital mortality (48% vs. 15%, *p* < 0.001) remained lower in the post-intervention group (Table 1).

### 3.2. Participation of Disciplines at Rounds

The intervention group consisted of two teams as mentioned above, the MICU APP team and the MICU Resident team. For the APP team, multidisciplinary participation in rounds increased significantly in the post-intervention period, increasing at the start of rounds from a median [IQR] number of disciplines of 5.0 [4.5, 5.0] to 7.0 [6.0, 8.0] (*p* = 0.003), as well as at the end of rounds from 5.0 [4.0, 6.0] to 7.0 [6.0, 8.0] (*p* = 0.002). Regarding the resident team, similar improvements were noted. At the start of rounds, the median [IQR] number of disciplines present increased from 6.0 [5.0, 7.0] to 7.0 [6.0, 8.0] (*p* = 0.006), while at the end of rounds the number of disciplines increased from 4.0 [4.0, 6.0] to 7.0 [6.0, 8.0] (*p* < 0.001). The NCCU team, which served as the control, demonstrated no significant change in multidisciplinary team member participation. At the start of rounds, the median [IQR] number of disciplines was 7.0 [6.5, 8.0] pre-intervention versus 7.0 [6.0, 8.0] post-intervention (*p* = 0.696) and at the conclusion of rounds, those numbers were 7.0 [6.0, 8.0] versus 6.0 [6.0, 7.0] (*p* = 0.183) (Table 1).

### 3.3. Duration of Rounds

The study noted significant improvement in rounding efficiency with the intervention. The mean ± SD duration of rounds decreased from 3.99 ± 1.00 h to 2.55 ± 0.70 h (*p* < 0.001). However, it also must be stated that the mean ± SD number of patients per day decreased from 18 ± 3 to 14 ± 4 (*p* < 0.001). Most notably, the median [IQR] time per patient decreased from 13.00 [12.00, 14.75] min to 10.30 [9.47, 12.00] min (*p* < 0.001), representing a 20.8% reduction in rounding time per patient.

### 3.4. Clinical Outcomes (Exploratory)

The physical therapy (PT) consultation rates increased significantly from the pre-intervention period to the post-intervention period (45.8% vs. 67.5%, *p* < 0.001). The median [IQR] time to PT consult decreased from 4 [1, 9] days pre-intervention to 2 [1, 4] days post-intervention (*p* = 0.001). Similarly, occupational therapy (OT) consultation rates increased from the pre-intervention to the post-intervention period (42.7% vs. 66.5%, *p* < 0.001), with the median [IQR] time to OT consult decreasing from 4 [2, 11] days to 2 [1, 4] days (*p* < 0.001). Delirium incidence decreased from the pre-intervention to the post-intervention period but did not attain statistical significance (29.0% vs. 22.0%, *p* = 0.155). Incidence of central venous catheter (CVC) use decreased from 58.0% pre-intervention to 25.0% post-intervention (*p* < 0.001), although among patients who had CVCs the median [IQR] duration remained similar (7 [3, 12] days vs. 6 [3, 12] days, *p* = 0.836). Arterial line placement rates decreased from 55.0% pre-intervention to 26.5% post-intervention (*p* < 0.001), with median [IQR] duration having decreased significantly from 5 [2, 8] days to 3 [1, 4] days (*p* = 0.001). Urinary catheter placement decreased from 75.6% pre-intervention to 58.0% post-intervention (*p* = 0.001). The median [IQR] duration of urinary catheterization decreased from 7 [3, 12] days pre-intervention to 4 [2, 9] days post-intervention (*p* = 0.162) (Table 1).

These data were further analyzed to account for the significant mortality difference between the pre- and post-intervention groups. An exploratory post hoc subgroup analysis was performed with survivors only. Among survivors (*n* = 66 pre-intervention and *n* = 169 post-intervention), there were sustained improvements noted in the post-intervention group with regard to the outcomes mentioned above. The median [IQR] time to PT consult improved from 4 [1, 11] days pre-intervention to 2 [1, 4] days post-intervention (*p* = 0.002), as did the median [IQR] time to OT consult from 4 [2, 12] days to 2 [1, 4] days (*p* < 0.001). Incidence of CVC rates dropped from 45.5% pre-intervention to 17.8% post-intervention (*p* < 0.001), as did the incidence of arterial lines (39.4% vs. 21.9%, *p* = 0.010), median [IQR] arterial catheter duration (5 [2, 8] days vs. 2 [1, 3] days, *p* = 0.003), and incidence of urinary catheters (69.7% vs. 51.5%, *p* = 0.008). No significant difference was noted in delirium incidence (30.3% pre-intervention vs. 20.7% post-intervention, *p* = 0.137) (Table 1).

## 4. Discussion

This single-center, pre/post quality improvement cohort study, featuring a concurrent nonintervention control group, found that implementing a standardized multidisciplinary rounding checklist incorporating the ICU Liberation bundle elements and FAST HUGS BID mnemonic, along with a real-time paging notification for rounding start time and location, was associated with a substantial increase in multidisciplinary participation for the two intervention group teams. The intervention was also significantly associated with increased efficiency of rounds (decreasing minutes spent per patient), and enhanced care-process measures, including earlier PT/OT consultation and reduced invasive device use. These results indicate that rounding processes can be altered to enhance the delivery of evidence-based care in the ICU.

The MICU APP and resident teams had significant increases in the number of disciplines participating in rounds at both the beginning and the end of rounds following implementation of the low-cost interventions of a paging system notification and a specific role-defined rounding process. There were no corresponding changes in the concurrent NCCU control, thus providing evidence that the changes were a result of the intervention and not because of some external factor. Prior literature has shown that inconsistencies in round start times, varying round locations, unclear role expectations, and inefficient communication have all been barriers to successful rounds in the ICU [2]. Conversely, established roles and checklists are known facilitators of successful rounds [2]. Brown and colleagues also reported improved attendance of nonphysician disciplines and reduced rounding time after standardization of rounding processes [3]. Our study adds to previous findings by demonstrating that the increases in participation were sustained throughout the entire rounds, confirmed against a control group, which is essential to developing reliable plans and completing handoffs for each patient [3].

The paging notification system provided a solution to a coordination problem in environments where multidisciplinary team members are shared among many different units. The absence of increased participation in the concurrent NCCU control supports that the combination of reliable notification and standardized structure was the likely contributor to the observed changes, rather than a broader institutional cultural shift. Notably, the NCCU group maintained a high baseline participation rate of seven disciplines throughout both study periods as these rounds were first in the daily sequence and maintained at least a defined daily starting time and relative location. It could also highlight that MICU teams required a systemic intervention to achieve optimal attendance and address the time and location barriers that exist between the teams.

The reduction in time per patient on rounds (20.8%) is equivalent to approximately 38 min of time per day for a typical census of 14 patients, or 5.7 h of labor per day across the nine disciplines ideally present for rounds. This equates to approximately 28.5 h per week (or 1482 h per year) or up to 185 eight-hour workdays saved throughout the year. Although the rounding duration may be affected by a lower average census in the post-intervention period, the reduction in time per patient is considered a more reliable indicator of productivity as it is less impacted by fluctuations in the daily census of the unit. Improved efficiency has been noted in multiple studies where similar redesigns of structured rounding have also included reduced redundant discussion, improved flow of care, and increased availability of required team members [3,7]. Additionally, the observed improvements in PT/OT usage and invasive device stewardship suggest that the intervention’s effect of shortening the duration of rounds did not negatively affect quality of care/clinical thoroughness but rather it facilitated consistent delivery of clinical best practices.

The significant decrease in median time to physical and occupational therapy consultation from 4 days to 2 days (*p* < 0.001) aligns with the ICU Liberation Campaign’s ABCDEF bundle, which defines early mobilization as physical activity initiated within 48 to 72 h of admission [4,8,9]. Higher bundle compliance correlates with improved patient outcomes, including decreased mortality, reduced need for mechanical ventilation, fewer ICU readmissions, and a dose-response reduction in delirium [4,8,10]. Prompt initiation of therapy is critical; combining early physical therapy with minimized sedation improves functional status at hospital discharge [11,12] and combats rapid neuromuscular dysfunction, which can cause up to 2% daily muscle mass loss during the first week of immobilization [13]. Similarly, substantial reductions in the rate of placement of central lines, arterial lines, and urinary catheters, along with shorter durations of arterial catheter use, all fit into the FAST HUGS BID framework and represent best practices for ICUs [14]. Approaches using daily goals can help avoid omitting best practices, create a common mental model among team members, and improve reliability when devices are removed [15]. A landmark Michigan Keystone ICU Study was the first to demonstrate that by implementing a daily assessment of whether the line was necessary as one component of a bundle of comprehensive interventions (the other components included handwashing and chlorhexidine skin preparation), median central line-associated blood stream infections reduced from 2.7 per 1000 catheter days to zero [16]. In our study, these effects persisted even after excluding non-survivors, indicating that the decreases in the number of devices placed were not solely due to differential mortality in the pre-intervention period. However, selection bias remains possible.

The range of diagnoses and degree of illness varied widely among those who were hospitalized in the mixed ICUs. The pre-period mortality rate in this cohort was also higher than in most reports on cohorts of mixed ICU patients, which indicates the potential influence of both case mix and time-period effects. The pre-intervention period was also the time of peak COVID-19 burden locally (15.3% of the cohort). Since we did not have information on the specific strains (e.g., occupancy rates, staffing ratios, delayed transfers) at the time of admission to the ICU, we cannot rule out the possibility that the increased mortality rates in this cohort were related to pandemic-related strain rather than the rounding intervention itself. A systematic review and meta-analysis of critically ill patients with COVID-19 reported in-hospital mortality rates ranging from 28 to 41 percent in 2020–2021 [17]; therefore, it is possible that the baseline mortality rate was also elevated in the current study due to the temporal context. It is possible that the resource limitations during the pandemic negatively affected the quality of care, and that the structured multidisciplinary communication during rounds partially mitigated the negative impact of such limitations; however, this is purely speculative without having direct measurements of care quality.

Prior randomized controlled trials investigating ICU checklist-based interventions have documented improvements in care processes without demonstrating a corresponding improvement in mortality [5]. The 2016 CHECKLIST-ICU trial evaluated the use of ICU checklist-based interventions in 6761 patients treated in 118 hospitals in Brazil and found no difference in mortality between treatment and control arms, although improved process measures were noted [5]. More recently, evidence derived from meta-analyses shows a more nuanced view of ICU rounding checklists. In 2024, MacKinnon and colleagues conducted a systematic review of 30 studies that investigated the relationship between ICU rounding checklists and in-hospital mortality; their analysis revealed that ICU rounding checklists were associated with lower in-hospital mortality (risk ratio = 0.80, 95% CI 0.70–0.92); however, the certainty of evidence was very low [18]. Therefore, the observed mortality effect in this study should be viewed as hypothesis-generating and supportive, albeit not definitive, of the notion that mortality benefits are mediated through the improvement of reliable evidence-based care processes.

In complex ICU environments, effective multidisciplinary collaboration is essential but difficult to execute reliably. In our institution, team members were frequently shared across multiple services, and rounds occurred at variable times and locations. This variability made it challenging for clinicians to consistently participate at the appropriate moment, even when motivated to do so. As a result, key input was sometimes delayed or omitted, care plans were fragmented, and additional follow-up communication was often required after rounds. These inefficiencies reflect a system-level problem: the reliability of information exchange rather than the quality of clinical decision-making itself. Prior work has identified inconsistent timing, unclear role expectations, and inefficient communication as major barriers to effective ICU rounds [2].

Importantly, the rounding checklist embedded evidence-based ICU care frameworks, including elements of the ABCDEF bundle and the FAST HUGS BID acronym, directly into the rounding workflow [1,4,14]. This design shifted best practices from being dependent on individual recall to being supported by system structure. In high-cognitive-load environments such as the ICU, this distinction is critical. The intervention therefore functioned not merely as a reminder tool, but as a reliability tool, facilitating consistent execution of high-value care behaviors. The observed improvements in multidisciplinary participation and rounding efficiency support the hypothesis that improving the reliability of team-based processes may be an important mechanism by which downstream improvements in care delivery are achieved.

Despite improvements in rounding participation, efficiency, and select process-of-care measures, the intervention was not associated with a statistically significant change in delirium outcomes. This finding is not unexpected. Delirium is a complex, multifactorial syndrome influenced by illness severity, sedation practices, sleep disruption, medication exposure, metabolic abnormalities, and baseline cognitive vulnerability [10]. In routine clinical practice, delirium assessment and documentation are also variable, which may further limit the sensitivity of retrospective analyses, and this was not a specific target of the intervention in our study. Additionally, the study may have been underpowered to detect modest changes in delirium, particularly if the primary effects of the intervention were upstream—improving communication, coordination, and process reliability—rather than directly modifying delirium-specific risk factors. Large observational studies of ABCDEF bundle implementation have demonstrated associations between bundle compliance and delirium reduction, but the magnitude of effect varies widely depending on implementation strategy and context [4,10].

Several limitations should be considered when interpreting this study’s findings. First, this was a single-center, retrospective study, which limits generalizability; it remains unknown if these findings would be reproducible in other types of intensive care units or in institutions with different staffing models. Furthermore, because the post-intervention data collection was limited to a specific time frame, it is unclear whether the observed improvements in multidisciplinary participation and rounding efficiency were sustained long-term beyond the study period. Second, there was a substantial time gap between the pre- and post-intervention periods, during which secular trends—including changes in case mix and pandemic-related strain—may have influenced outcomes. To partially mitigate this temporal confounding, we specifically adjusted our mortality analysis for COVID-19 status and performed a post hoc subgroup analysis of survivors to ensure our process-of-care improvements were not solely driven by differential mortality. Third, the severity of illness was not adjusted for using standardized scores such as SOFA or APACHE, which may have contributed to residual confounding. Unfortunately, given the retrospective study design and limited resources, manual calculation of these severity scores for the entire 331-patient cohort was deemed to be beyond the scope of our study.

Our study had several strengths. The inclusion of a concurrent nonintervention control group significantly bolsters internal comparison for participation outcomes and helps isolate the impact of the rounding intervention from broader institutional trends. This is highly valuable for pre-post quality improvement projects like ours. Our study goes beyond simply measuring rounding duration by evaluating tangible process-of-care clinical outcomes tied to established evidence-based frameworks such as the ICU Liberation bundle and the FAST HUGS BID mnemonic (such as improved time to PT/OT consultation and reduced utilization of invasive devices). Our statistical approach also strengthens these findings; by adjusting for COVID-19 status and conducting a rigorous post hoc subgroup analysis of survivors, we demonstrated that the continued reductions in device utilization and time to therapy were improvements in care delivery rather than mere artifacts of differential mortality. Finally, our intervention itself, which happens to be a standardized checklist and a paging notification system, is inherently low-cost, scalable, and highly feasible to implement within existing critical care staffing structures.

Several analytic limitations should be acknowledged. Standardized illness severity scores like SOFA or APACHE were not available for all patients, limiting adjustment for baseline acuity. Time-to-consultation analyses did not formally account for death or discharge as competing events. Although a concurrent control group was used to assess multidisciplinary participation, it was not applied to patient-level outcomes, limiting causal inference. Pre- and post-intervention participation data were collected prospectively by the pharmacist, which may have introduced documentation bias but did ensure consistency. Additionally, intervention fidelity was not formally measured; therefore, we are unable to assess how consistently the rounding checklist was completed in its entirety or how reliably the paging system was used. Prior checklist-based ICU trials have similarly demonstrated improvements in process measures without consistent mortality benefits [5]. Clinical outcomes were exploratory and should be interpreted cautiously given multiple comparisons and residual confounding variables. The absence of multivariate adjustment in this regard is an important limitation. A survivor-only sensitivity analysis may introduce selection bias. Finally, longer-term outcomes such as functional recovery, post-ICU cognitive status, and quality of life were not evaluated.

Future studies could incorporate risk-adjusted analyses and more robust designs, such as interrupted time series or difference-in-differences approaches. Measuring fidelity metrics—such as checklist completion rates and real-time paging adherence—would allow for stronger mechanistic interpretation. Further work should also focus on downstream outcomes more closely aligned with the intervention’s proposed mechanisms, including ventilator days, sedation exposure, device utilization, infections, and ICU-acquired complications [4,8,11,12,13].

## Figures and Tables

**Figure 1:**
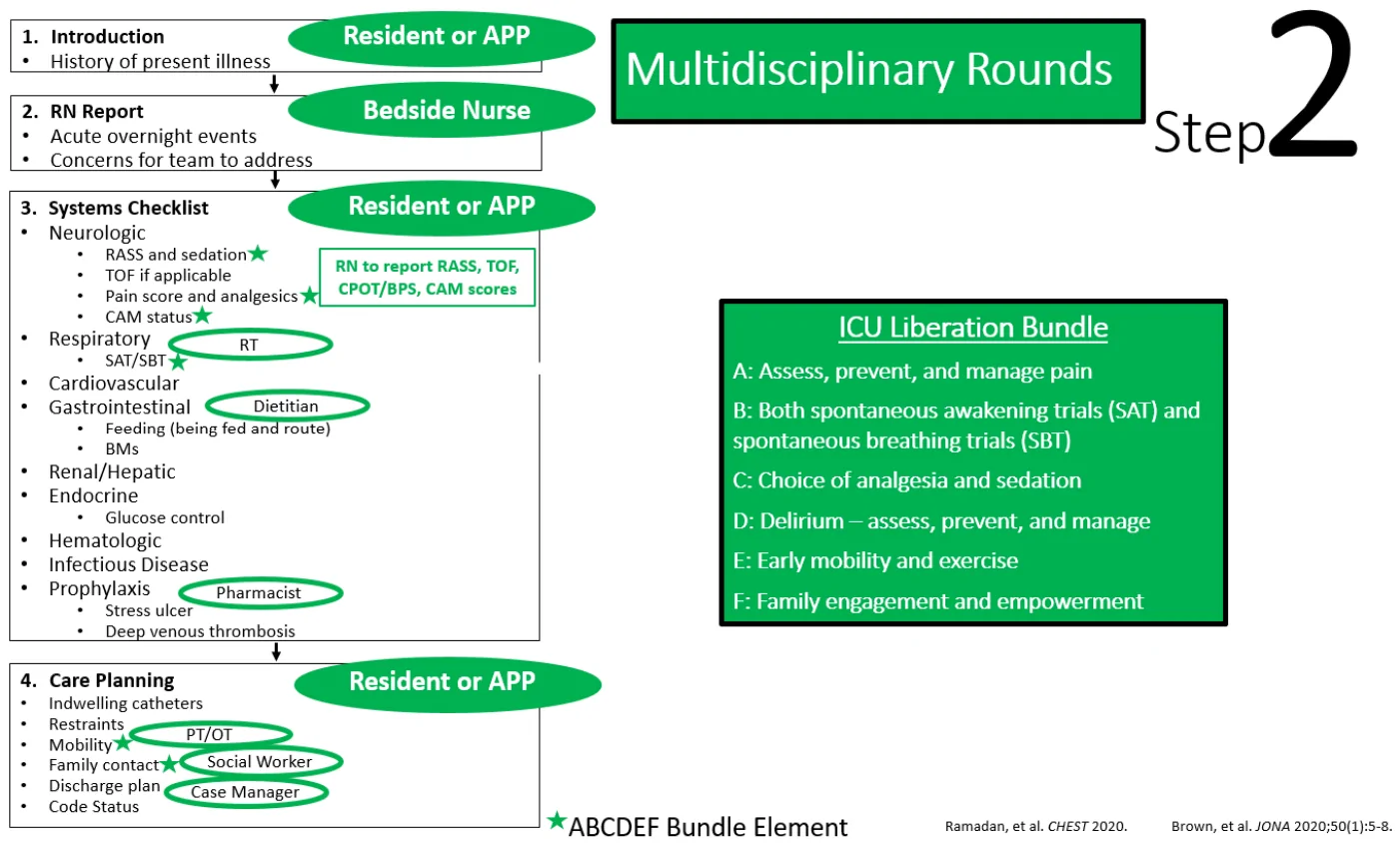
The standardized multidisciplinary rounding checklist/card intervention incorporating ICU Liberation (ABCDEF) bundle elements and the FAST HUGS BID mnemonic [3,6].

**Table 1:** Pre- and post-intervention rounding characteristics, full-cohort outcomes, and post hoc hospital-survivor analysis. All available values are included below.

Characteristic or Outcome	Pre-Intervention	Post-Intervention	*p* Value
**A. Rounding characteristics and participation**	***n* = 33 days**	***n* = 34 days**	
Patients per day	18 ± 3	14 ± 4	<0.001
Duration of rounds, hours	3.99 ± 1.00	2.55 ± 0.70	<0.001
Time per patient, minutes	13.00 [12.00, 14.75]	10.30 [9.47, 12.00]	<0.001
MICU APP team: disciplines at start of rounds	5.0 [4.5, 5.0]	7.0 [6.0, 8.0]; *n* = 31	0.003
MICU APP team: disciplines at end of rounds	5.0 [4.0, 6.0]	7.0 [6.0, 8.0]; *n* = 31	0.002
MICU resident team: disciplines at start of rounds	6.0 [5.0, 7.0]	7.0 [6.0, 8.0]	0.006
MICU resident team: disciplines at end of rounds	4.0 [4.0, 6.0]	7.0 [6.0, 8.0]	<0.001
NCCU control team: disciplines at start of rounds	7.0 [6.5, 8.0]	7.0 [6.0, 8.0]	0.696
NCCU control team: disciplines at end of rounds	7.0 [6.0, 8.0]	6.0 [6.0, 7.0]	0.183
**B. Full-cohort characteristics and exploratory outcomes**	***N* = 131 patients**	***N* = 200 patients**	
Age, years	65 [50, 75]	63 [52, 75]	0.631
COVID-19	20 (15.3%)	10 (5.0%)	0.003
ICU length of stay, days	2 [1, 6]	2 [1, 3]	0.200
ICU mortality	47 (35.9%)	22 (11.0%)	<0.001
In-hospital mortality	65 (49.6%)	31 (15.5%)	<0.001
PT consultation	60 (45.8%)	135 (67.5%)	<0.001
Time to PT consultation, days	4 [1, 9]; *n* = 50	2 [1, 4]; *n* = 112	0.001
OT consultation	56 (42.7%)	133 (66.5%)	<0.001
Time to OT consultation, days	4 [2, 11]; *n* = 48	2 [1, 4]; *n* = 114	<0.001
Delirium	38 (29.0%)	44 (22.0%)	0.155
Central venous catheter placed	76 (58.0%)	50 (25.0%)	<0.001
Summative CVC duration, days	7 [3, 12]; *n* = 44	6 [3, 12]; *n* = 34	0.836
Arterial catheter placed	72 (55.0%)	53 (26.5%)	<0.001
Summative arterial catheter duration, days	5 [2, 8]; *n* = 48	3 [1, 4]; *n* = 50	0.001
Urinary catheter placed	99 (75.6%)	116 (58.0%)	0.001
Summative urinary catheter duration, days	7 [3, 12]; *n* = 66	4 [2, 9]; *n* = 99	0.162
**C. Post hoc hospital-survivor analysis**	***N* = 66 patients**	***N* = 169 patients**	
PT consultation	44 (66.7%)	121 (71.6%)	0.466
Time to PT consultation, days	4 [1, 11]; *n* = 40	2 [1, 4]; *n* = 119	0.002
OT consultation	43 (65.2%)	119 (70.4%)	0.441
Time to OT consultation, days	4 [2, 12]; *n* = 39	2 [1, 4]; *n* = 104	<0.001
Delirium	20 (30.3%)	35 (20.7%)	0.137
Central venous catheter placed	30 (45.5%)	30 (17.8%)	<0.001
Summative CVC duration, days	10 [4, 19]; *n* = 24	4 [3, 11]; *n* = 25	0.107
Arterial catheter placed	26 (39.4%)	37 (21.9%)	0.010
Summative arterial catheter duration, days	5 [2, 8]; *n* = 24	2 [1, 3]; *n* = 37	0.003
Urinary catheter placed	46 (69.7%)	87 (51.5%)	0.008
Summative urinary catheter duration, days	4 [2, 14]; *n* = 39	4 [2, 9]; *n* = 82	0.892

Abbreviations: APP, advanced practice provider; CVC, central venous catheter; ICU, intensive care unit; MICU, medical ICU; NCCU, neurovascular critical care unit; OT, occupational therapy; PT, physical therapy.

## Data Availability

The corresponding author shall respond to queries regarding raw data, which is available on request.

## Abbreviations

The following abbreviations are used in this manuscript:

ABCDEF	Assessment, Prevention and Management of Pain; Both Spontaneous Awakening and Spontaneous Breathing trials; Choice of Analgesia and Sedation; Delirium Assessment, Prevention and Management; Early Mobility and Exercise, Family Engagement and Empowerment
FAST HUGS BID	Feeding, Analgesia, Sedation, Thromboprophylaxis, Head of bed elevation, Ulcer prophylaxis, Glycemic control, Spontaneous breathing trial, Bowel care, Indwelling catheters, De-escalation of antibiotics/non-essential drugs
